# Circumsporozoite-Specific T Cell Responses in Children Vaccinated with RTS,S/AS01_E_ and Protection against *P falciparum* Clinical Malaria

**DOI:** 10.1371/journal.pone.0025786

**Published:** 2011-10-06

**Authors:** Ally Olotu, Philippe Moris, Jedidah Mwacharo, Johan Vekemans, Domtila Kimani, Michel Janssens, Oscar Kai, Erik Jongert, Marc Lievens, Amanda Leach, Tonya Villafana, Barbara Savarese, Kevin Marsh, Joe Cohen, Philip Bejon

**Affiliations:** 1 Kenya Medical Research Institute/ Wellcome Trust Programme, Centre for Geographic Medicine Research, Coast, Kilifi, Kenya; 2 GlaxoSmithKline Biologicals, Rixensart, Belgium; 3 PATH Malaria Vaccine Initiative (MVI), Bethesda, Maryland, United States of America; 4 MedImmune, LLC, Gaithersburg, Maryland, United States of America; 5 Centre for Clinical Vaccinology and Tropical Medicine, Nuffield Department of Medicine, University of Oxford, Oxford, United Kingdom; Menzies School of Health Research, Australia

## Abstract

**Background:**

RTS,S/AS01**_E_** is the lead candidate pre-erythrocytic malaria vaccine. In Phase IIb field trials the safety profile was acceptable and the efficacy was 53% (95%CI 31%–72%) for protecting children against clinical malaria caused by *P. falciparum*. We studied CS-specific T cell responses in order to identify correlates of protection.

**Methods and Findings:**

We used intracellular cytokine staining (for IL2, IFNγ, and TNFα), *ex-vivo* ELISPOTs (IFNγ and IL2) and IFNγ cultured ELISPOT assays to characterize the CS-specific cellular responses in 407 children (5–17 months of age) in a phase IIb randomized controlled trial of RTS,S/AS01_E_ (NCT00380393). RTS,S/ AS01_E_ vaccinees had higher frequencies of CS-specific CD4+ T cells producing IFNγ, TNFα or IL2 compared to control vaccinees. In a multivariable analysis TNFα^+^ CD4^+^ T cells were independently associated with a reduced risk for clinical malaria among RTS,S/AS01_E_ vaccinees (HR = 0.64, 95%CI 0.49–0.86, p = 0.002). There was a non-significant tendency towards reduced risk among control vaccinees (HR = 0.80, 95%CI 0.62–1.03, p = 0.084), albeit with lower CS-specific T cell frequencies and higher rates of clinical malaria. When data from both RTS,S/AS01**_E_** vaccinees and control vaccinees were combined (with adjusting for vaccination group), the HR was 0.74 (95%CI 0.62–0.89, p = 0.001). After a Bonferroni correction for multiple comparisons (n-18), the finding was still significant at p = 0.018. There was no significant correlation between cultured or *ex vivo* ELISPOT data and protection from clinical malaria. The combination of TNFα^+^ CD4^+^ T cells and anti-CS antibody statistically accounted for the protective effect of vaccination in a Cox regression model.

**Conclusions:**

RTS,S/AS01**_E_** induces CS-specific Th1 T cell responses in young children living in a malaria endemic area. The combination of anti-CS antibody concentrations titers and CS-specific TNFα^+^ CD4^+^ T cells could account for the level of protection conferred by RTS,S/AS01**_E_**. The correlation between CS-specific TNFα^+^ CD4^+^ T cells and protection needs confirmation in other datasets.

## Introduction

RTS,S is the lead candidate pre-erythrocytic malaria vaccine [1]. The vaccine antigen consists of 19 copies of the central tandem repeats and C-terminal region of the *P. falciparum* circumsporozoite protein (CS) fused to hepatitis B surface antigen (HBsAg), and co-expressed with unfused HBsAg in *Saccharomyces cerevisiae* cells. The two proteins spontaneously assemble in the yeast cells to form virus-like particles. The RTS,S antigen has been tested with two different alternative Adjuvant Systems: AS02 or AS01. Both Adjuvant Systems contain the immunostimulants monophosphoryl lipid A (MPL®) and QS21, formulated either with an oil-in-water emulsion (AS02) or with liposomes (AS01).

Formulated in either Adjuvant System, the RTS,S antigen induces high concentrations of anti-circumsporozoite protein (CS) antibodies [2], [3], [4], [5], [6], [7]. Correlations between anti-CS concentrations and protection against infection were statistically significant on experimental challenge with *P. falciparum* in malaria naïve adults [7], of borderline significance on natural challenge of semi-immune adults [4], and significant on natural challenge of children in a malaria endemic area [8]. Anti-CS titers did not correlate with protection against clinical malaria episodes in children [4], [9], but we recently identified a non-linear relationship between concurrent (rather than peak) anti-CS titers and protection from clinical malaria in children [10].

CD4^+^ T cell responses to pre-erythrocytic antigens prevent intra-hepatocytic parasites developing in both human and mouse studies [11], [12]. Potential mechanisms include TNFα induced apoptosis [13] or inhibition of parasite growth [14] and IFNγ induced NO production [15]. RTS,S-induced cell mediated immune responses have been assessed using proliferation assays, cytokine production on cell culture, intracellular cytokine staining and flow-cytometry, and *ex-vivo* and cultured ELISPOT assays [16], [17].

RTS,S/AS immunization induces a CD4^+^ T cell response but little or no detectable CD8^+^ T cell response [7], [18], [19], [20], [21]. Sun et al observed IFNγ-producing CD8+ T cells, but only after cells were stimulated for 10–14 days *in vitro* [22]. Barbosa et al reported CD8+ T cell responses after 42 hours *in vitro* stimulation on comparing RTS,S/AS02 vaccinees with control vaccinees at 10 weeks, but not at 4 weeks, post immunization [23].

The frequency of poly-functional CD4+ T cells identified by intracellular cytokine staining (ICS) correlated with protection from *P. falciparum* infection after experimental challenge in adults [7], [24]. In a field study, Reece et al reported a correlation between protection against re-infection and cultured IFNγ ELISPOT assays using a single conserved T cell epitope from the CS protein [20]. However, this analysis was not adjusted for anti-CS titers, and did not include ICS studies. A borderline correlation between single cytokine ICS results and protection from *P. falciparum* infection was shown in a field study in infants [23].

In order to examine associations with protection against clinical malaria, we assessed the CS-specific cellular immune responses in 447 children using ICS, *ex vivo* IFNγ and IL2 ELISPOT, and cultured IFNγ ELISPOT assays in a phase II b randomized clinical trial of RTS,S/AS01**_E_** versus control, in which we observed 53% (95%CI 31%–72%) protection against clinical malaria [25]. The blood volumes sampled in children prevented us from using an ICS assay previously reported in adult studies [7], but a whole blood ICS assay requiring smaller blood volumes has been developed and used in two phase II trials in Ghana [26] and Gabon [27]. These studies showed that the vaccine induced CD4+ IL2, TNFα or IFNγ producing cells, but CD40L was not detectable using the whole blood assay for children in Sub-Saharan Africa. We therefore did not include CD40L staining in the assay for our study.

The qualification of correlates of immunity and surrogates of protection has been recently reviewed [28], [29]. The Prentice criteria require that: a) vaccination predicts protection; b) vaccination predicts the potential surrogate; c) the surrogate predicts protection among vaccinees and d) that the surrogate accounts for all the effect of vaccination [30]. If vaccination is an independent predictor of outcome after including the potential surrogate in the analysis, this suggests that other mechanisms are involved. On the other hand, if including the potential surrogate in analysis removes vaccination as a predictor, this is consistent with the effect of vaccination being mediated by the surrogate marker.

## Methods

The study protocol and its amendments received ethical and scientific approval from Kenya Medical Research Institute National Ethics Committee, National Institute for Medical Research of Tanzania, the Oxford Tropical Research Ethics Committee, the London School of Hygiene and Tropical Medicine Ethics committee and the Western Institutional Review Board in Seattle. The study was conducted in accordance with the Helsinki Declaration of 1964 (revised 1996) and Good Clinical Practice guidelines and was overseen by an Independent data monitoring committee and local safety monitors. Written informed consent was obtained using approved Swahili or Giriama consent forms. Illiterate parents thumb printed the consent form which was countersigned by an independent, literate witness.

We conducted a randomized controlled trial to evaluate the efficacy and safety of RTS,S/AS01**_E_** against clinical malaria episodes due to *P. falciparum* infection in Kilifi, Kenya and Korogwe, Tanzania. There were 894 children between the two sites, of which the 447 children enrolled in Kilifi were assessed for vaccine induced cellular immunity using ICS and ELISPOT.

Details on randomization, immunization and surveillance have been published previously [25]. In Kilifi, 447 children 5–17 months old were randomized and received either RTS,S/AS01**_E_** or rabies vaccine in a 1∶1 ratio according to 0, 1, 2 month schedule. Both vaccines were given intramuscularly in the left deltoid. The primary end point was clinical malaria, defined as the presence of fever (axillary temperature ≥37.5°C) and *P. falciparum* parasitaemia ≥2500/µL. Active and passive surveillance for malaria was conducted by field workers and study personnel at local dispensaries.

Children were vaccinated between March and August 2007. Blood was taken for immunological studies before vaccination, one month post dose 3, then on March 2008 irrespective of the time of recruitment (i.e. between 4 and 10 months post dose 3, mean 8 months), 12 months post dose 3 and in October 2008 irrespective of time of recruitment, (i.e. between 12 and 18 months post dose 3, mean 15 months). Peak malaria transmission was between May and August 2008.

### CS antibody measurement

Antibodies to the *P. falciparum* circumsporozoite protein (CS) tandem repeat epitope were assessed by ELISA at the Center for Vaccinology, Ghent University Hosptial, Belgium. Results were reported in EU/mL. Plates were adsorbed with the recombinant antigen R32LR that contained the sequence [NVDP(NANP)15]2LR [31].

### Peptides

A set of 32 15-mer, peptides were used, overlapping by 11 amino acids to cover the full length of the CS antigen used in the vaccine (3D7 strain). All these peptides were used in a single pool for the ICS studies, but they were divided into three pools for ELISPOT studies, namely; a) the conserved region including the NANP repeats, b) the variant TH2R region and c) the variant TH3R region and conserved CS.T3T region (Table 1).

**Table 1:** Peptide pools.

Peptide	Sequence
NANP and conserved region peptides pool	
Pept 1	MMAP DPNANPNANPN
Pept 2	NANP NANPNANPNAN
Pept 3	DPNA NPNANPNKNNQ
Pept 4	NPNA NPNKNNQGNGQ
Pept 5	NPNK NNQGNGQGHNM
Pept 6	NNQG NGQGHNMPNDP
Pept 7	NGQG HNMPNDPNRNV
Pept 8	HNMP NDPNRNVDENA
Pept 9	NDPN RNVDENANANS
Pept 10	RNVD ENANANSAVKN
Pept 11	ENAN ANSAVKNNNNE
TH2R region peptides pool	
Pept 12	ANSA VKNNNNEEPSD
Pept 13	VKNN NNEEPSDKHIK
Pept 14	NNEE PSDKHIKEYLN
Pept 15	PSDK HIKEYLNKIQN
Pept 16	HIKE YLNKIQNSLST
Pept 17	YLNK IQNSLSTEWSP
Pept 18	IQNS LSTEWSPCSVT
Pept 19	LSTE WSPCSVTCGNG
TH3R/CS.T3T region peptides pool	
Pept 20	WSPC SVTCGNGIQVR
Pept 21	SVTC GNGIQVRIKPG
Pept 22	GNGI QVRIKPGSANK
Pept 23	QVRI KPGSANKPKDE
Pept 24	KPGS ANKPKDELDYA
Pept 25	ANKP KDELDYANDIE
Pept 26	KDEL DYANDIEKKIC
Pept 27	DYAN DIEKKICKMEK
Pept 28	DIEK KICKMEKCSSV
Pept 29	KICK MEKCSSVFNVV
Pept 30	MEKC SSVFNVVNSSI
Pept 31	KCSS VFNVVNSSIGL

All three peptide pools were combined for the ICS assay. The pools were used separately for the *ex vivo* and plating out of the cultured ELISPOT assay.

### ELISPOT assays

Peripheral blood mononuclear cells (PBMC) were separated and incubated in RPMI medium (Sigma-Aldrich) with 10% Human AB serum. We used Millipore MAIP S45 plates and MabTech antibodies for ELISPOT assays according to the manufacturer's instructions. For *ex vivo* ELISPOT assays (IFNγ and IL2), 2×10^5^ per well of freshly isolated PBMCs were incubated in 100µl final volume at 2.5 µg/ml circumsporozoite antigen peptides (see table 1) for 18–20 hours before developing the plates. The positive and negative controls were 20 µg/ml Phytohaemaglutinin (Sigma-Aldrich) and media alone, respectively. For cultured ELISPOTs, 1x10^6^ PBMC were incubated in 0.5 mls of 10µg/ml/peptide of pooled peptides in a 24-well plate. On days 3 and 7, 250µl of culture supernatant was replaced with 250µl culture medium containing 20 IU/ml recombinant IL 2. On day 9, the cells were washed three times and left overnight before an ELISPOT assay (IFNγ only) was done according to the method used for *ex vivo* ELISPOTs. Spot forming cell numbers were counted by ELISPOT plate reader (Autoimmun Diagnostika, version 3.0).

### ELISPOT analysis

ELISPOT wells were assayed in duplicate, and the final result was the mean of two wells. The negative control well result was subtracted from each peptide well. ELISPOTs failed quality control if the negative control well had more than 25 spots or the positive control had less than 50 spots. The results from the three peptide pools were added to calculate total responses. Results are presented as number of spots per million incubated PBMC.

### Whole blood ICS assay

Whole blood was stimulated in Kilifi within 2 hours of being drawn. 350 µl of whole blood plus 100 µl of phosphate buffered saline (PBS) was incubated in three different 15 ml Falcon tube, with 1 µg/ml of anti-CD28 anti-CD49d monoclonal antibodies (supplied by BD). After 2 hours, Brefeldin A was added to a final concentration of 1 µg/ml and incubation was continued overnight at 37°C ± 1- CO_2_ 5 to 7%. EDTA was then added to a final concentration at 5 mM, and after 15 minutes 1 ml FACS lysing solution (BD). The positive control was stimulated using staphylococcal enterotoxin B (SEB) and negative control was PBS without peptides. Circumsporozoite antigen peptides were added to the third tube to a final concentration of 1 µg/ml (see Table 1). The cells were then washed in PBS and re-suspended in PBS with 10% DMSO and stored at –70°C for transport to GSK in Rixensart. In GSK, cells were thawed, washed and stained with alexa-fluor 700 conjugated anti-CD3 (Pharmingen), peridinin-chlorophyll (PerCP)-conjugated anti-CD4 (BD Biosciences) and allophycocyanin (APC)-H7 conjugated anti-CD8 antibodies (BD Biosciences). Cells were fixed and permeabilized using the Cytofix/Cytoperm buffer kit (Pharmingen), and stained with APC conjugated anti-IL-2 (Pharmingen), fluorescein-isothiocyanate (FITC)-conjugated anti-IFN-γ (Pharmingen) and phycoerythrin (PE) cyanin–7 (Cy7)-conjugated anti-TNFα (Pharmingen). Cells were washed, re-suspended in fetal-calf-serum (FCS)-containing phosphate buffered saline (PBS) and analyzed on a BD™ LSR II flow cytometer (BD Biosciences). Events were counted using the automatic gating on the FACSDiva software (BD Biosciences). Conventional rules were used to gate on single cells, then the lymphocyte subset based on forward and side scatter. CD3 and CD4/CD8 positive cells and then cytokine expression was classified into positive/negative using FACSDiva software. An example of the output with gating shown is given in Supporting Information S1. We required at least 10,000 CD4 + events and 5,000 CD8+ events. Acquisition was stopped when 75,000 CD4+ events had been acquired, and we acquired more than 50,000 CD4+ events for the majority of samples (>90%).

Results from antigen-stimulated cultures were not excluded from analysis on the basis of positive/negative control results, in the absence of established criteria. Data are represented as background subtracted CS-specific events per million CD4+ or CD8+ T cells.

Assays were conducted according to sample availability, since blood samples were limited to 5 mls. In order of priority, the assays conducted were; ICS, IFNγ *ex vivo* ELISPOT, IL2 *ex vivo* ELISPOT and cultured ELISPOT. Samples were processed within 3 hours of being taken. ICS samples were stored for 3 to 4 months at –70°C before staining. The samples were processed during the double-blind phase of the study.

### Statistical analysis

Geometric mean responses are calculated and a Student's T test was performed on log-transformed values to compare between vaccination groups. A paired T test on log-transformed values was used to compare time-courses, and correlations between assays were examined using Pearson's product moment calculation on log-transformed values. Cox regression for the primary endpoint (clinical malaria with *P. falciparum* density ≥2500/µL) was adjusted for age at first vaccination, village, distance from the health facility, bed net use and anti-circumsporozoite (CS) antibody levels by dichotomizing concurrent anti-CS titers at 40 EU/mL [10]. Cellular responses were analyzed as time-varying covariates, applying the result from the time of the most recent clinic visit. A Bonferroni correction was subsequently calculated for the independently significant explanatory variables. Responses were log transformed to produce normal distributions before inclusion in the Cox regression models. Analysis was conducted on the According To Protocol vaccinees. STATA version 10 was used.

## Results

Blood samples were processed from 407 children. Data were acquired from 1,066 ICS assays (from three different clinic visits), 660 cultured ELISPOTs (from four different clinic visits), 780 *ex vivo* ELISPOTs for IFNγ (from three clinic visits) and 453 *ex vivo* ELISPOTs for IL2 production (from 3 clinic visits). 56 (8%), 12 (2%) and 21 (5%) assays failed quality control criteria for positive and negative controls for cultured, and *ex vivo* IFNγ and *ex vivo* IL2 ELISPOTs, respectively. For ICS assay, the results from the positive control were at least 100 cells per million above the negative control for 1045 (98.0%), 1057 (99.1%) and 1055 (99.0%) for IFNγ, IL2 and TNFα, respectively.

**Figure 1:**
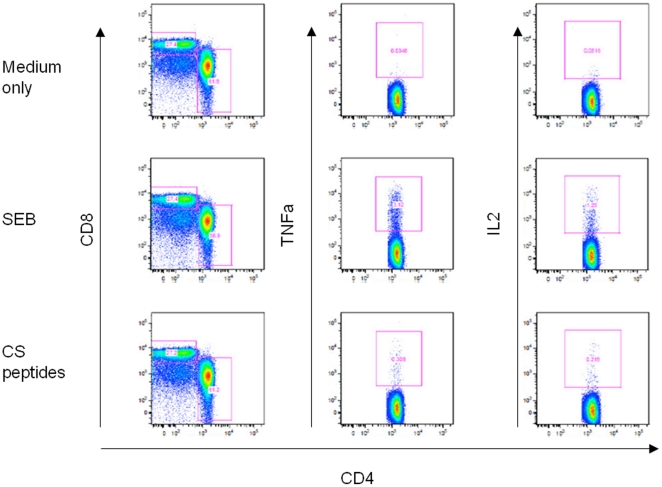
An example plot of FACS data acquired following intra-cellular cytokine staining is shown for negative control (medium only), positive control (i.e. staphylococcal enterotoxin B, SEB) and CS peptides.

The geometric mean responses to the negative control were 75, 165 and 159 cells per million for IFNγ, IL2 and TNFα ICS results respectively, and average responses to positive control were 3,768, 18,895 and 3,454 cells per million. The mean responses to CS antigen vary by timepoint and by vaccination group, but the ranges were 11 to 25, 10 to 681 and 8 to 426 for IFNγ, IL2 and TNFα, respectively. There was no variation in responses to control by time point (p = 0.15, p = 0.15, p = 0.6) or by vaccination group at the first timepoint post vaccination (p = 0.4, p = 0.36 and p = 0.39). An example of the flow cytometry analysis is shown in Figure 1.

### Vaccine induced anti-CS T cell responses: ICS assays

**Figure 2:**
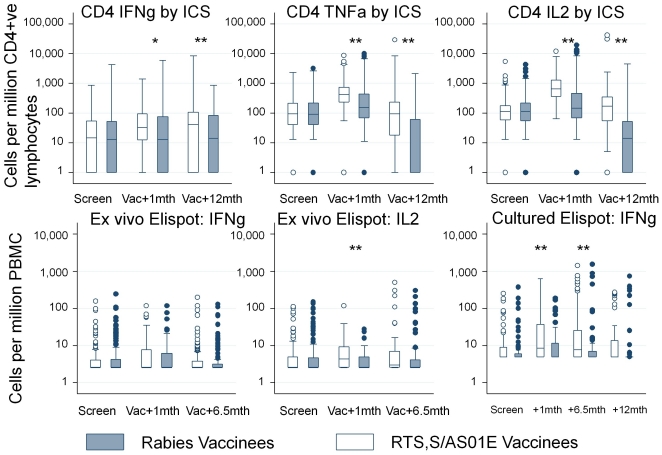
The time course of anti-CS CD4+ ICS responses and summed ELISPOT responses is shown per time point for RTS,S/AS01_E_ and control vaccination groups. * indicates p<0.05 and ** indicates p<0.005.

CD4+ and CD8+ anti-CS T cell responses were detected in both vaccination groups using ICS. There were no significant differences between the groups pre-vaccination. Vaccination with RTS,S/AS01_E_ induced CD4+ but no CD8+ anti-CS T cell responses. The strongest responses were seen for IL2 producing CD4 T cells at one month post vaccination (a mean of 681 cells per million, 95%CI 585–792), followed by TNFα (426 cells per million, 95%CI 362–502), and weak IFNγ responses (25 cells per million, 95%CI 18–34) (Table 2, Figure 2). These levels corresponded to 3.2 fold, 2.3 fold and 1.9 fold increases for IL2, TNFα and IFNγ, respectively.

**Table 2:** Geometric means of CMI assays by clinic visit and by vaccination group.

Visit	Rabies		RTS,S/AS01_E_		
	Mean (95%CI)	N	Mean (95%CI)	N	p
**ICS: CD4+ve cells: IFNg**					
Screen	11(8–14)	197	12(9–16)	182	0.6
Vac+1 mth	13(10–18)	182	25(18–34)	170	0.01*
Vac+12 mths	11(7–15)	167	20(14–29)	168	0.009**
**ICS: CD4+ve cells: IL2**					
Screen	103(84–127)	197	94(76–117)	182	0.52
Vac+1 mth	212(183–245)	182	681(585–792)	170	2×10–13**
Vac+12 mths	10(7–14)	167	102(73–142)	168	9×10–19**
**ICS: CD4+ve cells: TNFa**					
Screen	86(69–108)	197	81(64–102)	182	0.69
Vac+1 mth	182(156–214)	182	426(362–502)	170[Table-fn nt102]	1×10–08**
Vac+12 mths	8(6–11)	167	48(34–68)	168	6×10–12**
**ICS: CD8+ve cells: IFNg**					
Screen	12(9–17)	178	7(5–10)	175	0.028#
Vac+1 mth	19(12–28)	168	19(12–28)	156	0.99
Vac+12 mths	10(6–15)	137	11(7–17)	127	0.73
**ICS: CD8+ve cells: IL2**					
Screen	156(128–191)	178	190(155–233)	175	0.24
Vac+1 mth	200(164–246)	168	215(175–266)	156	0.64
Vac+12 mths	9(6–13)	137	20(13–32)	127	0.004**
**ICS: CD8+ve cells: TNFa**					
Screen	49(36–68)	178	59(43–81)	175	0.43
Vac+1 mth	133(106–167)	168	162(127–205)	156	0.26
Vac+12 mths	15(10–24)	137	16(10–25)	127	0.91
**IFNg cultured ELISPOT: NANP and conserved region**					
Screen	27(21–35)	72	33(26–43)	70	0.22
Vac+1 mth	32(27–38)	86	28(24–32)	109	0.26
Vac+6.5 mths	29(25–35)	82	26(22–30)	122	0.34
Vac+12 mths	31(23–42)	55	27(21–36)	64	0.54
**IFNg cultured ELISPOT: TH2R region**					
Screen	33 (26–43)	72	36 (28–48)	70	0.66
Vac+1 mth	34(25–47)	86	66(50–88)	109	3×10–4**
Vac+6.5 mths	30(22–41)	83	60(47–78)	122	2×10–4**
Vac+12 mths	26(20–34)	55	39(30–51)	64	0.023*
**IFNg cultured ELISPOT: TH3R/ CS.T3T region**					
Screen	31 (23–41)	72	37 (28–48)	70	0.38
Vac+1 mth	30(23–40)	86	55(43–70)	109	3×10–4**
Vac+6.5 mths	32(23–44)	83	58(45–76)	122	0.003**
Vac+12 mths	33(24–46)	55	36(26–48)	64	0.79
**IFNg cultured ELISPOT: All CS peptides summed**					
Screen	75 (58–97)	72	90 (69–117)	70	0.7
Vac+1 mth	88(66–117)	86	151(117–195)	109	0.002**
Vac+6.5 mths	81(59–110)	82	145(113–187)	122	0.002**
Vac+12 mths	83(59–115)	55	104(76–141)	64	0.33
**IFNg ex vivo ELISPOT: NANP and conserved region**					
Screen	15(13–17)	152	14(12–16)	137	0.59
Vac+1 mth	15(13–18)	100	16(13–19)	104	0.83
Vac+6.5 mths	13(12–14)	145	12(11–13)	142	0.21
**IFNg ex vivo ELISPOT: TH2R region**					
Screen	15(13–17)	152	14(12–16)	137	0.56
Vac+1 mth	16(13–19)	100	18(15–22)	104	0.23
Vac+6.5 mths	14(13–16)	145	14(13–17)	142	0.96
**IFNg ex vivo ELISPOT: TH3R/ CS.T3T region**					
Screen	18(15–20)	152	17(15–20)	137	0.79
Vac+1 mth	19(15–23)	100	21(17–25)	104	0.43
Vac+6.5 mths	13(11–15)	144	15(13–17)	141	0.23
**IFNg ex vivo ELISPOT: All CS peptides summed**					
Screen	40(35–46)	152	39(34–45)	137	0.69
Vac+1 mth	44(36–54)	100	48(40–59)	104	0.5
Vac+6.5 mths	35(31–40)	144	35(31–40)	141	0.94
**IL2 ex vivo ELISPOT: NANP and conserved region**					
Screen	15(13–18)	107	15(12–18)	89	0.8
Vac+1 mth	12(10–14)	62	14(12–17)	56	0.11
Vac+6.5 mths	15(12–20)	76	15(11–21)	63	0.98
**IL2 ex vivo ELISPOT: TH2R region**					
Screen	16(13–19)	107	16(13–20)	89	0.93
Vac+1 mth	13(10–17)	62	21(16–28)	56	0.003**
Vac+6.5 mths	18(14–23)	76	17(13–22)	63	0.76
**IL2 ex vivo ELISPOT: TH3R/ CS.T3T region**					
Screen	17(15–21)	107	17(14–21)	89	0.9
Vac+1 mth	15(12–19)	62	24(19–31)	56	0.003**
Vac+6.5 mths	18(13–24)	76	29(20–41)	62	0.022*
**IL2 ex vivo ELISPOT: All peptides summed**					
Screen	44(37–53)	107	42(34–51)	89	0.71
Vac+1 mth	35(27–45)	62	56(44–73)	56	0.002**
Vac+6.5 mths	45(34–60)	76	53(39–72)	62	0.43

*for p<0.05 or ** for p<0.005 where Mean for Rabies group < Mean for RTS,S/AS01_E_ group. # for p<0.05 or ## for p<0.005 where Mean for Rabies group > Mean for RTS,S/AS01_E_ group.

### ELISPOT assays

Three different peptide pools were used for the ELISPOT assays, allowing a more detailed analysis of immunogenicity. Cultured ELISPOT results were higher among RTS,S/AS01**_E_** vaccinees than among rabies vaccinees at 1 month and 6.5 months post vaccination, but not at 12 months. IFNγ *ex vivo* ELISPOT results did not vary by vaccination group at any timepoint. IL2 *ex vivo* ELISPOT responses were significantly higher in RTS,S/AS01**_E_** vaccinees at 1 month post vaccination, but not at 6.5 months (Table 2) compared with rabies vaccinees.

**Figure 3:**
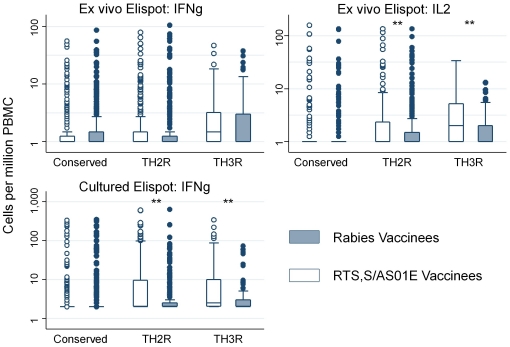
ELISPOT responses are shown for the individual stimulating peptide pools at 1 month post vaccination with RTS,S/AS01_E_.

For both the cultured IFNγ ELISPOT and *ex vivo* IL2 ELISPOT, the vaccine induced cellular responses were limited to two peptide pools (i.e. TH2R and TH3R/CS.T3T pools, Table 1). No responses were detected to the third peptide pool (NANP and conserved region peptides; Figure 3).

### Time course of responses (ICS assays)

The frequencies of IL2, TNFα and IFNγ producing CD4+ T cells by ICS was significantly higher at one month after the final vaccination with RTS,S/AS01**_E_** compared with pre-vaccination levels (p<0.0001, p<0.0001, p = 0.0006, respectively). There was then a fall in responses between 1 month and 12 months post vaccination, falling to pre-vaccination levels for IL2 (p<0.0001) and TNFα (p<0.0001). IFNγ producing CD4+ T cells remained above pre-vaccination levels, albeit at low frequency throughout. However, there was an even more pronounced fall in CD4+ T cell responses among control vaccinees (Table 2), and so RTS,S/AS01**_E_** vaccinees had substantially higher T cell responses than control vaccinees at 12 months post vaccination (p<0.0001 for TNFα and IL2, p = 0.009 for IFNγ).

### Inter assay correlations

There were strong correlations between the different cytokines detected by ICS, and also between IL2/IFNγ ELISPOT results one month after vaccination with RTS,S/AS01**_E_** (Table 3). Cultured ELISPOTs were significantly associated with ICS results, but not with *ex vivo* ELISPOT results (IFNγ or IL2). Antibody titres were associated with all the cellular assays except *ex vivo* IFNγ ELISPOTs (Table 3).

**Table 3:** Inter-assay Correlation coefficients of CMI assays at 1 month post vaccination with RTS,S/AS01_E_.

	CD4+ IFNγ	CD4+ IL2	CD4+ TNFα	Antibody (CS)	Cultured IFNγ	IFNγ ELISPOT	IL2 ELISPOT
CD4+ IFNγ	1						
CD4+ IL2	**0.38** ***	1					
CD4+ TNFα	**0.32** ***	**0.66** ***	1				
Antibody (CS)	**0.14** ***	**0.35** ***	**0.26** ***	1			
Cultured IFNγ	**0.14** **	**0.15** **	**0.18** ***	**0.22** ***	1		
IFNγ ELISPOT	−0.05	−0.05	0.02	0.03	−0.02	1	
IL2 ELISPOT	0	0.01	0.15*	0.11*	−0.06	**0.31** ***	1

* = p<0.05.

** = p<0.001.

*** = p<0.0001.

### Correlates of immunity

After vaccination with RTS,S/AS01**_E,_** an increasing frequency of TNFα producing, CS-specific CD4+ cells detected using ICS was associated with a reduced risk of clinical malaria (HR = 0.64 for each 10 fold increase in the frequency of CD4+ TNFα+ T cells, 95%CI 0.49–0.86, p = 0.002). On ICS, IFNγ production by CS-specific CD4+ T cells was associated with a reduced risk of clinical malaria of borderline significance (p = 0.07, Table 4). TNFα and IFNγ producing CS-specific CD4+ T cells were at much lower frequencies among control vaccinees, but nevertheless were associated with reduced risks of clinical malaria of borderline significance. When data from both RTS,S/AS01**_E_** vaccinees and control vaccinees were combined (with adjusting for vaccination group), the overall hazard ratios were 0.74 (95%CI 0.62–0.89, p = 0.001) and 0.79 (95%CI 0.67–0.94, p = 0.007) for TNFα and IFNγ, respectively. On Bonferroni adjustment, these p values were 0.018 and 0.13, respectively. Similar results were observed when adjusting for anti-CSP antibody titres as a continuous variable (HR = 0.75, 95%CI 0.62–0.91, p = 0.003 with p = 0.054 after Bonferroni adjustment, and HR = 0.81, 95%CI 0.68–0.95, p = 0.01 with p = 0.13 after Bonferroni adjustment for TNFα CD4+ T cells and IFNγ CD4+ T cells, respectively).

**Table 4:** The hazard ratio from Cox regression models (with 95% CI) for the outcome clinical malaria by CMI assays.

	Both datasets		Rabies Vaccinees		RTS,S/AS01_E_ Vaccinees	
Assay	HR (95%CI)	p	HR (95%CI)	p	HR (95%CI)	p
ICS: CD4 cells						
**IFN**γ	**0.79(0.67**–**0.94)**	**0.007**	**0.81(0.66**–**1.01)**	**0.058**	**0.77(0.59**–**1.02)**	**0.07**
IL2	0.9(0.76–1.07)	0.23	0.97(0.78–1.22)	0.81	0.77(0.55–1.08)	0.13
**TNF**α	**0.74(0.62**–**0.89)**	**0.001**	**0.8(0.62**–**1.03)**	**0.084**	**0.64(0.49**–**0.86)**	**0.002**
ICS: CD8 cells						
IFNγ	1.07(0.91–1.25)	0.43	1.13(0.93–1.37)	0.21	0.93(0.68–1.27)	0.65
IL2	0.85(0.69–1.05)	0.13	0.92(0.7–1.21)	0.56	0.79(0.56–1.12)	0.19
TNFα	0.87(0.72–1.04)	0.12	0.83(0.66–1.05)	0.11	0.93(0.68–1.28)	0.66
IFNγ cultured ELISPOT						
NANP	1.01(0.65–1.57)	0.95	0.9(0.54–1.52)	0.7	1.08(0.44–2.68)	0.87
TH2R	0.76(0.51–1.14)	0.18	0.67(0.3–1.52)	0.34	0.79(0.49–1.27)	0.33
TH3R	0.94(0.67–1.32)	0.72	0.99(0.61–1.61)	0.97	0.86(0.53–1.39)	0.54
Sum	0.95(0.67–1.34)	0.77	0.92(0.54–1.57)	0.77	0.92(0.58–1.47)	0.73
IFNγ *ex vivo* ELISPOT						
NANP	1.61(1.01–2.55)	0.044	1.54(0.87–2.72)	0.14	1.38(0.62–3.08)	0.44
TH2R	1(0.59–1.69)	1	0.9(0.44–1.85)	0.78	1.1(0.49–2.45)	0.81
TH3R	1.62(1.04–2.52)	0.032	1.57(0.82–2.99)	0.17	1.4(0.75–2.64)	0.29
Sum	1.35(0.86–2.12)	0.2	1.32(0.73–2.4)	0.35	1.23(0.61–2.47)	0.57
IL2 *ex vivo* ELISPOT						
NANP	1.18(0.56–2.51)	0.67	1(0.29–3.39)	0.99	1.38(0.46–4.17)	0.57
TH2R	0.57(0.23–1.45)	0.24	0.49(0.13–1.79)	0.28	0.81(0.2–3.2)	0.76
TH3R	0.94(0.45–1.97)	0.87	0.73(0.21–2.49)	0.61	0.97(0.39–2.42)	0.94
Sum	0.83(0.38–1.83)	0.65	0.81(0.25–2.56)	0.72	0.78(0.23–2.62)	0.69

HR =  Hazard Ratio for each log (i.e. ten-fold) increase in frequency of T cells. Confidence intervals are 5–95%. HRs are adjusted by anti-CS antibody titre (in 2 groups), age, area of residence, ITN use and distance from the dispensary. NANP = NANP and conserved region peptides pool, TH2R = TH2R region peptides pool, TH3R = TH3R and CS.T3T region peptide pool, Sum =  all three peptide pools summed.

**Figure 4:**
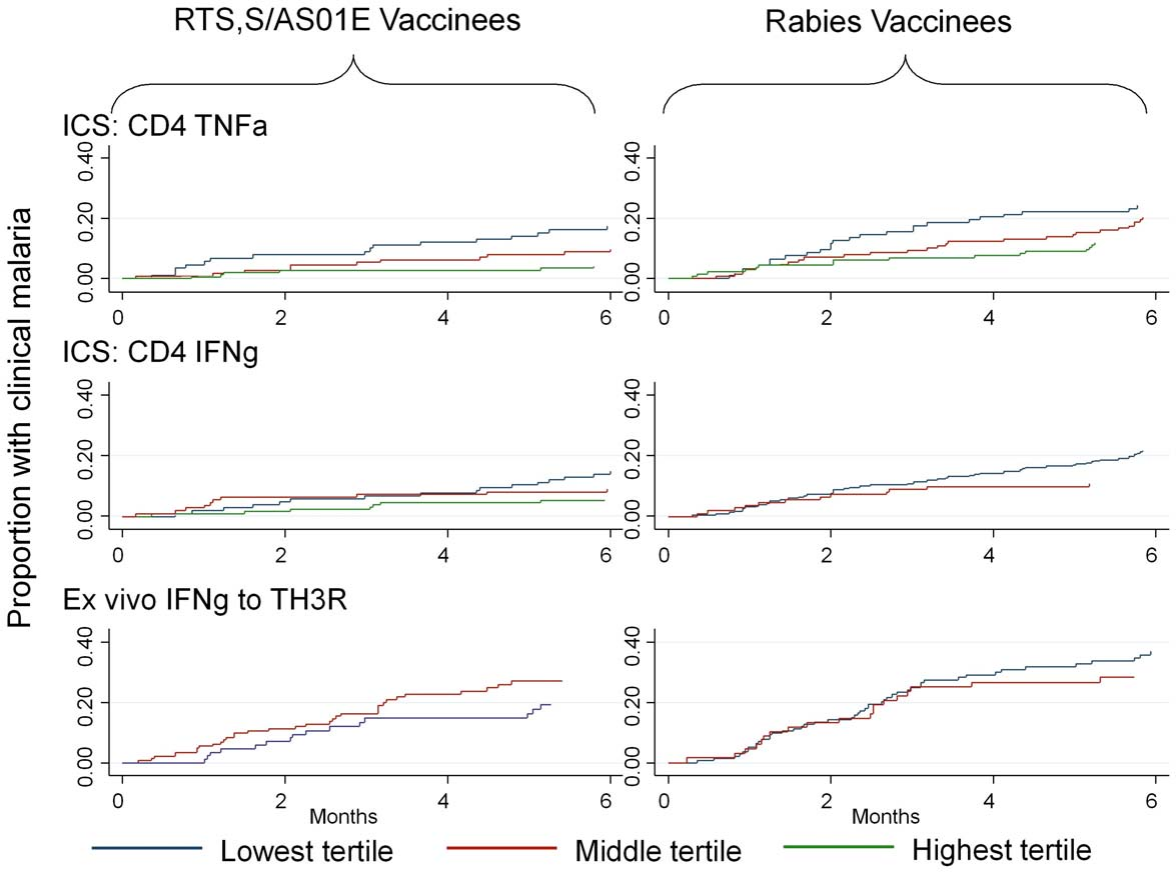
Survival plots with time to first episode of clinical malaria plotted for RTS,S/AS01E (left columns) and control vaccinees (left and right columns) according to tertile of CD4+, TNFα responses (top row), CD4+ IFNγ responses (middle row) and IFNγ *ex vivo* ELISPOT responses to TH3R/CS.T3T peptides pool (lower row). Where more than one third of responses were at the lower limit of detection, the lower two tertiles are combined (and hence only 2 tertiles are displayed on some plots). For CD4+ TNFα+ responses, the tertiles were 1 to 154 (lower), 155 to 407 (middle) and 408 to 28,840 (upper) cells per million for RTS,S/AS01E vaccinees, and 1 to 26 (lower), 27 to 165 (middle) and 166 to 10,000 (upper) cells per million for control vaccinees. For CD+ IFNγ+ responses the tertiles were 1 to 12 (lower), 13 to 66 (middle) and 67 to 8,320 (upper) cells per million for RTS,S/AS01E vaccinees, and 1 to 40 (lower) and 41 to 5,980 (upper) cells per million for rabies vaccinees. The time point “0 months” refers to the time of a blood draw. Cellular responses were analyzed as time-varying covariates, where the effect of cellular responses from all available blood draws was related to clinical malaria episodes during the period of monitoring after each measurement. Therefore, each RTS,S vaccinee could contribute to 2 periods of monitoring. These three assays were selected for the figure because significant associations on Cox regression were seen (Table 4).

In order to display the effect graphically, the cellular responses were split by tertile (Figure 4). The middle tertiles for TNFα are at intermediate risk, suggesting a continuous change in risk as the frequency of TNFα cells increases rather than a threshold effect.

When the frequencies of TNFα producing CD4+ T cells and IFNγ producing CD4+ T cells were combined in the same model, TNFα CD4+ T cell frequency was the independent factor (i.e. HR = 0.76, 95%CI 0.64–0.89, p = 0.001 compared with HR = 0.84, 95%CI 0.71–1.00, p = 0.050 for the frequency of IFNγ producing CD4+ T cells). An interaction term generated by multiplying the frequency of TNFα CD4+ T cells by antibody concentrations was not significant in determining risk (HR = 0.79, 95%CI 0.51–1.2, p = 0.29).

On applying the fourth of the Prentice criteria, we found that vaccination group was still an independent predictor of clinical malaria risk in a multivariable model including CD4+ TNFα+ cells (HR = 0.69, 95%CI 0.48–0.97, p = 0.036). In other words, only 42% of the effect of vaccination could be accounted for by CD4+ TNFα+ cells. However, when anti-CS titers were added to the model the effect of vaccine became non-significant (HR = 0.93, 95%CI 0.62–1.42, p = 0.76, i.e. 87% of the effect of vaccination was accounted for).

Hence, while neither CD4+ TNFα+ cells nor anti-CS antibodies alone accounted for all of the effect of vaccination with RTS,S/AS01_E_ on clinical malaria risk, the combination of CD4+ TNFα+ T cells and anti-CS antibodies together could account for all of the statistical effect of vaccination.

## Discussion

Vaccination with RTS,S/AS01_E_ induced circumsporozoite protein (CS) specific T cell responses in 5–17 month-old children living in a malaria endemic area. The frequency of CD4+ TNFα+ T cells on ICS was associated with protection from clinical malaria. Although the use of 15-mer peptides may have been sub-optimal to demonstrate CD8+ T cell responses, we did in fact identify both CD4+ and CD8+ responses above negative control conditions for both RTS,S/AS01_E_ and control vaccinees. However, the CD8+ responses were apparently not induced by vaccination, and presumably are the result of exposure to malaria parasites [32].

If TNFα producing CD4+ T cells are causally related to protection, they should be associated with protection whether they are acquired by vaccination or by natural exposure to malaria parasites. Indeed, a borderline statistical correlation between TNFα+ CD4+ T cells and a reduced risk of clinical malaria was observed among control vaccinees. However, the overall level of protection afforded by T cells among RTS,S/AS01_E_ vaccinees was greater, since the vaccinated children had 2–3 fold more TNFα+ CD4+ T cells at 1 month and 8–10 fold more at 12 months post vaccination. The frequency of TNFα_CD4+ T cells at 12 months post vaccination was below screening (i.e. pre-vaccination) levels, despite the fact that clinical protection was sustained at 15 months [10]. Since clinical protection is determined by contemporaneous comparison with control vaccinees, it is therefore theoretically possible that the relative difference between RTS,S/AS01_E_ vaccinees and control vaccines is relevant despite this fall.

T cell responses to CSP rose between screening and 1 month post-vaccination, and then fell to lower levels at 12 months post vaccination. This was seen in both ELISPOT and ICS studies, and could not be explained by greater background responses (which did not change over time and were subtracted from antigen-specific responses), or by non-specific responses detected in changing positive controls over time. The temporary increase in CSP specific responses parallels the increase and decrease in antibodies to blood stage antigens seen in the same children [33]. Antibody responses made by young children to blood stage antigens are often short-lived [34], and may reflect short-term changes in exposure [35]. Taking together the antibody data, the consistent pattern in ICS and ELISPOT studies, the stability of positive and negative control responses, and the accounting for background reactivity by subtracting negative control responses from antigen-specific responses, show no significant variation over time for positive and negative controls, we conclude that the T cell responses are raised by exposure to malaria during the transmission season, but are short-lived and therefore not sustained once exposure falls.

We did not identify an association between protection and T cell responses detected by the cultured IFNγ assay, as previously reported [20]. However, we tested pools of peptides rather than individual peptides, and the previously reported association was specific to the CS.T3T peptide. The CS.T3T peptide was contained in a pool of TH3R/CS.T3T peptides. In other studies, the CS.T3T peptide accounted for more than half of the overall response seen in the TH3R/CS.T3T peptide pool [36]. Furthermore, we identified very little IFNγ production in our study. Previous studies showing marked IFNγ production have been done in adults [18], and IFNγ production may be suppressed in children in malaria endemic areas [37].

*Ex vivo* ELISPOT studies did not correlate with ICS studies for the same cytokines, even though both use overnight stimulations, and ICS results were 10-fold higher than ELISPOT results. This may be partially explained by measuring ELISPOT assays per million PBMC, whereas ICS is measured per million CD4+ T cells. However, *ex vivo* and cultured ELISPOTs identify different cell populations, the latter more closely reflecting a central memory phenotype [38], [39], [40]. Hence it is possible that ICS and cultured ELISPOT identify central memory cells, but *ex vivo* ELISPOTs identify effector phenotypes. ELISPOT assays failing internal control standards were excluded, but similar control standards were not pre-defined for ICS data. However, this applied to a minority of assays (2% and 5%, respectively for *ex vivo* IFNγ and IL2 ELISPOTS) which did not correlate with ICS data, and 8% for cultured ELISPOTS, which did correlate. Hence it seems unlikely that lack of quality control standards for ICS explained the lack of correlation.

The CD4+ T cell response associated with protection in our analysis (i.e. TNFα production) were at a low frequency (mean 426 cells per million CD4 cells at peak). Higher frequency responses have been required to achieve protection in sporozoite challenge studies [7]. However, the antibody concentrations associated with protection are also higher in sporozoite challenge studies [7], [10]. These differences in outcome may be explained by the greater sporozoite inoculum used in challenge studies compared with exposure in the field [41]. The IFNγ response that was apparently associated with protection in our study was very low frequency and barely above the limit of detection (25 cells per million), and the apparent association is likely to reflect the association between IFNγ and TNFα rather than an independent effect. We were unable to assess polyfunctionality in the present study, because the high number of samples (n = 1066, with three conditions per sample) required an automated gating strategy using FACSDiva software (BD Biosciences). The FACSdiva analysis used for this study did not include Boolean gates in order to obtain polyfunctional T cell data. Further analysis to determine polyfunctionality in this large dataset is ongoing.

Multiple comparisons (i.e. 18) have been undertaken to identify the association between TNFα producing CD4+ T cells and protection from clinical malaria. However, the association was highly significant (p = 0.001) and remains significant after a Bonferroni correction (p = 0.018).

The Prentice criteria have been proposed as a way of qualifying surrogate endpoints [42], [43] and include four criteria [30]. We found that the combination of anti-CS titers and TNFα producing CD4+ T cells met all the criteria (i.e. vaccination was associated with protection; anti-CS titers and CD4+ TNFα+ T cells were both independently associated with vaccination; were both independently associated with protection; and the combination of anti-CS titers and CD4+ TNFα+ T cells, but not either alone, could account for the effect of vaccination in multi-variable Cox regression analysis). We found no significant interaction between anti-CS titers and TNFα+ T cells.

Microheterogeneity of malaria exposure has been observed in Kilifi [44], and may confound the association between antibodies to blood stage malaria antigens and the risk of malaria [33]. However, this is unlikely to explain the association between CD4+ TNFα+ T cells and protection from clinical malaria for two reasons: The direction of confounding was in the opposite direction in this cohort (i.e. microheterogeneity led to a confounded association between increasing antibody levels and increasing risk of malaria rather than protection), and the association with protection is more marked in RTS,S/AS01_E_ vaccinees rather than control vaccinees.

There is strong evidence that anti-CS antibodies inhibit sporozoite invasion [45], supporting a causal relationship, and TNFα may reduce the parasite's intrahepatocytic development [13], [14]. However, it is possible that the frequency of CD4+ TNFα+ T cells is associated with another causal mediator of immunity (for instance better quality antibody responses, enhanced T cell memory or polyfunctionality). These further characterizations of the immune response should now be a priority, since establishing an immunological surrogate endpoint will accelerate the development of candidate malaria vaccines, inform monitoring the persistence of immune responses and inform the timing of a booster dose.

## Supporting Information

Supporting Information S1 (PPT)

## References

[1] Ballou WR (2009). The development of the RTS,S malaria vaccine candidate: challenges and lessons.. Parasite Immunol.

[2] Lell B, Agnandji S, von Glasenapp I, Haertle S, Oyakhiromen S (2009). A randomized trial assessing the safety and immunogenicity of AS01 and AS02 adjuvanted RTS,S malaria vaccine candidates in children in Gabon.. PLoS One.

[3] Bojang KA, Olodude F, Pinder M, Ofori-Anyinam O, Vigneron L (2005). Safety and immunogenicty of RTS,S/AS02A candidate malaria vaccine in Gambian children.. Vaccine.

[4] Bojang KA, Milligan PJ, Pinder M, Vigneron L, Alloueche A (2001). Efficacy of RTS,S/AS02 malaria vaccine against Plasmodium falciparum infection in semi-immune adult men in The Gambia: a randomised trial.. Lancet.

[5] Doherty JF, Pinder M, Tornieporth N, Carton C, Vigneron L (1999). A phase I safety and immunogenicity trial with the candidate malaria vaccine RTS,S/SBAS2 in semi-immune adults in The Gambia.. Am J Trop Med Hyg.

[6] Stoute JA, Slaoui M, Heppner DG, Momin P, Kester KE (1997). A preliminary evaluation of a recombinant circumsporozoite protein vaccine against Plasmodium falciparum malaria. RTS,S Malaria Vaccine Evaluation Group.. N Engl J Med.

[7] Kester KE, Cummings JF, Ofori-Anyinam O, Ockenhouse CF, Krzych U (2009). Randomized, double-blind, phase 2a trial of falciparum malaria vaccines RTS,S/AS01B and RTS,S/AS02A in malaria-naive adults: safety, efficacy, and immunologic associates of protection.. J Infect Dis.

[8] Guinovart C, Aponte JJ, Sacarlal J, Aide P, Leach A (2009). Insights into long-lasting protection induced by RTS,S/AS02A malaria vaccine: further results from a phase IIb trial in Mozambican children.. PLoS One.

[9] Alonso PL, Sacarlal J, Aponte JJ, Leach A, Macete E (2004). Efficacy of the RTS,S/AS02A vaccine against Plasmodium falciparum infection and disease in young African children: randomised controlled trial.. Lancet.

[10] Olotu A, Lusingu J, Leach A, Lievens M, Vekemans J (2011). Efficacy of RTS,S/AS01E malaria vaccine and exploratory analysis on anti-circumsporozoite antibody titres and protection in children aged 5-17 months in Kenya and Tanzania: a randomised controlled trial.. Lancet Infect Dis.

[11] Wang R, Charoenvit Y, Corradin G, De La Vega P, Franke ED (1996). Protection against malaria by Plasmodium yoelii sporozoite surface protein 2 linear peptide induction of CD4+ T cell- and IFN-gamma-dependent elimination of infected hepatocytes.. J Immunol.

[12] McConkey SJ, Reece WH, Moorthy VS, Webster D, Dunachie S (2003). Enhanced T-cell immunogenicity of plasmid DNA vaccines boosted by recombinant modified vaccinia virus Ankara in humans.. Nat Med.

[13] Butler NS, Schmidt NW, Harty JT (2010). Differential effector pathways regulate memory CD8 T cell immunity against Plasmodium berghei versus P. yoelii sporozoites.. J Immunol.

[14] Depinay N, Franetich JF, Gruner AC, Mauduit M, Chavatte JM (2011). Inhibitory effect of TNF-alpha on malaria pre-erythrocytic stage development: influence of host hepatocyte/parasite combinations.. PLoS ONE.

[15] Doolan DL, Hoffman SL (2000). The complexity of protective immunity against liver-stage malaria.. J Immunol.

[16] Casares S, Brumeanu TD, Richie TL (2010). The RTS,S malaria vaccine.. Vaccine.

[17] Moorthy VS, Ballou WR (2009). Immunological mechanisms underlying protection mediated by RTS,S: a review of the available data.. Malar J.

[18] Lalvani A, Moris P, Voss G, Pathan AA, Kester KE (1999). Potent induction of focused Th1-type cellular and humoral immune responses by RTS,S/SBAS2, a recombinant Plasmodium falciparum malaria vaccine.. J Infect Dis.

[19] Stoute JA, Kester KE, Krzych U, Wellde BT, Hall T (1998). Long-term efficacy and immune responses following immunization with the RTS,S malaria vaccine.. J Infect Dis.

[20] Reece WH, Pinder M, Gothard PK, Milligan P, Bojang K (2004). A CD4(+) T-cell immune response to a conserved epitope in the circumsporozoite protein correlates with protection from natural Plasmodium falciparum infection and disease.. Nat Med.

[21] Pinder M, Reece WH, Plebanski M, Akinwunmi P, Flanagan KL (2004). Cellular immunity induced by the recombinant Plasmodium falciparum malaria vaccine, RTS,S/AS02, in semi-immune adults in The Gambia.. Clin Exp Immunol.

[22] Sun P, Schwenk R, White K, Stoute JA, Cohen J (2003). Protective immunity induced with malaria vaccine, RTS,S, is linked to Plasmodium falciparum circumsporozoite protein-specific CD4+ and CD8+ T cells producing IFN-gamma.. J Immunol.

[23] Barbosa A, Naniche D, Aponte JJ, Manaca MN, Mandomando I (2009). Plasmodium falciparum-specific cellular immune responses after immunization with the RTS,S/AS02D candidate malaria vaccine in infants living in an area of high endemicity in Mozambique.. Infect Immun.

[24] Olotu AI, Fegan G, Bejon P (2010). Further analysis of correlates of protection from a phase 2a trial of the falciparum malaria vaccines RTS,S/AS01B and RTS,S/AS02A in malaria-naive adults.. J Infect Dis.

[25] Bejon P, Lusingu J, Olotu A, Leach A, Lievens M (2008). Efficacy of RTS,S/AS01E vaccine against malaria in children 5 to 17 months of age.. N Engl J Med.

[26] Ansong D, Asante KP, Vekemans J, Owusu SK, Owusu R (2011). T cell responses to the RTS,S/AS01(E) and RTS,S/AS02(D) malaria candidate vaccines administered according to different schedules to Ghanaian children.. PLoS ONE.

[27] Agnandji ST, Fendel R, Mestre M, Janssens M, Vekemans J (2011). Induction of Plasmodium falciparum-specific CD4+ T cells and memory B cells in Gabonese children vaccinated with RTS,S/AS01(E) and RTS,S/AS02(D).. PLoS ONE.

[28] Plotkin SA (2008). Vaccines: correlates of vaccine-induced immunity.. Clin Infect Dis.

[29] Qin L, Gilbert PB, Corey L, McElrath MJ, Self SG (2007). A framework for assessing immunological correlates of protection in vaccine trials.. J Infect Dis.

[30] Prentice RL (1989). Surrogate endpoints in clinical trials: definition and operational criteria.. Stat Med.

[31] Macete EV, Sacarlal J, Aponte JJ, Leach A, Navia MM (2007). Evaluation of two formulations of adjuvanted RTS, S malaria vaccine in children aged 3 to 5 years living in a malaria-endemic region of Mozambique: a Phase I/IIb randomized double-blind bridging trial.. Trials.

[32] Flanagan KL, Lee EA, Gravenor MB, Reece WH, Urban BC (2001). Unique T cell effector functions elicited by Plasmodium falciparum epitopes in malaria-exposed Africans tested by three T cell assays.. J Immunol.

[33] Bejon P, Cook J, Bergmann-Leitner E, Olotu A, Lusingu J (2011). Effect of the Pre-erythrocytic Candidate Malaria Vaccine RTS,S/AS01E on Blood Stage Immunity in Young Children.. J Infect Dis.

[34] Kinyanjui SM, Bull P, Newbold CI, Marsh K (2003). Kinetics of antibody responses to Plasmodium falciparum-infected erythrocyte variant surface antigens.. J Infect Dis.

[35] Bejon P, Turner L, Lavstsen T, Cham G, Olotu A (2011). Serological Evidence of Discrete Spatial Clusters of Plasmodium falciparum Parasites.. PLoS ONE.

[36] Dunachie SJ, Walther M, Vuola JM, Webster DP, Keating SM (2006). A clinical trial of prime-boost immunisation with the candidate malaria vaccines RTS,S/AS02A and MVA-CS.. Vaccine.

[37] Bejon P, Mwacharo J, Kai O, Todryk S, Keating S (2007). The induction and persistence of T cell IFN-gamma responses after vaccination or natural exposure is suppressed by Plasmodium falciparum.. J Immunol.

[38] Godkin AJ, Thomas HC, Openshaw PJ (2002). Evolution of epitope-specific memory CD4(+) T cells after clearance of hepatitis C virus.. J Immunol.

[39] Keating SM, Bejon P, Berthoud T, Vuola JM, Todryk S (2005). Durable Human Memory T Cells Quantifiable by Cultured Enzyme-Linked Immunospot Assays Are Induced by Heterologous Prime Boost Immunization and Correlate with Protection against Malaria.. J Immunol.

[40] Todryk SM, Pathan AA, Keating S, Porter DW, Berthoud T (2009). The relationship between human effector and memory T cells measured by ex vivo and cultured ELISPOT following recent and distal priming.. Immunology.

[41] Bejon P, Andrews L, Andersen RF, Dunachie S, Webster D (2005). Calculation of liver-to-blood inocula, parasite growth rates, and preerythrocytic vaccine efficacy, from serial quantitative polymerase chain reaction studies of volunteers challenged with malaria sporozoites.. J Infect Dis.

[42] Kohberger RC, Jemiolo D, Noriega F (2008). Prediction of pertussis vaccine efficacy using a correlates of protection model.. Vaccine.

[43] Ray ME, Bae K, Hussain MH, Hanks GE, Shipley WU (2009). Potential surrogate endpoints for prostate cancer survival: analysis of a phase III randomized trial.. J Natl Cancer Inst.

[44] Bejon P, Williams TN, Liljander A, Noor AM, Wambua J (2010). Stable and unstable malaria hotspots in longitudinal cohort studies in Kenya.. Plos Med.

[45] Hollingdale MR, Appiah A, Leland P, do Rosario VE, Mazier D (1990). Activity of human volunteer sera to candidate Plasmodium falciparum circumsporozoite protein vaccines in the inhibition of sporozoite invasion assay of human hepatoma cells and hepatocytes.. Trans R Soc Trop Med Hyg.

