# Supplementary figures and images for: Circumsporozoite-Specific T Cell Responses in Children Vaccinated with RTS,S/AS01E and Protection against P falciparum Clinical Malaria
Source: PLoS One. 2011 Oct 6;6(10):e25786. doi: 10.1371/journal.pone.0025786 (PMC3188575; doi:10.1371/journal.pone.0025786)

## Slide 1
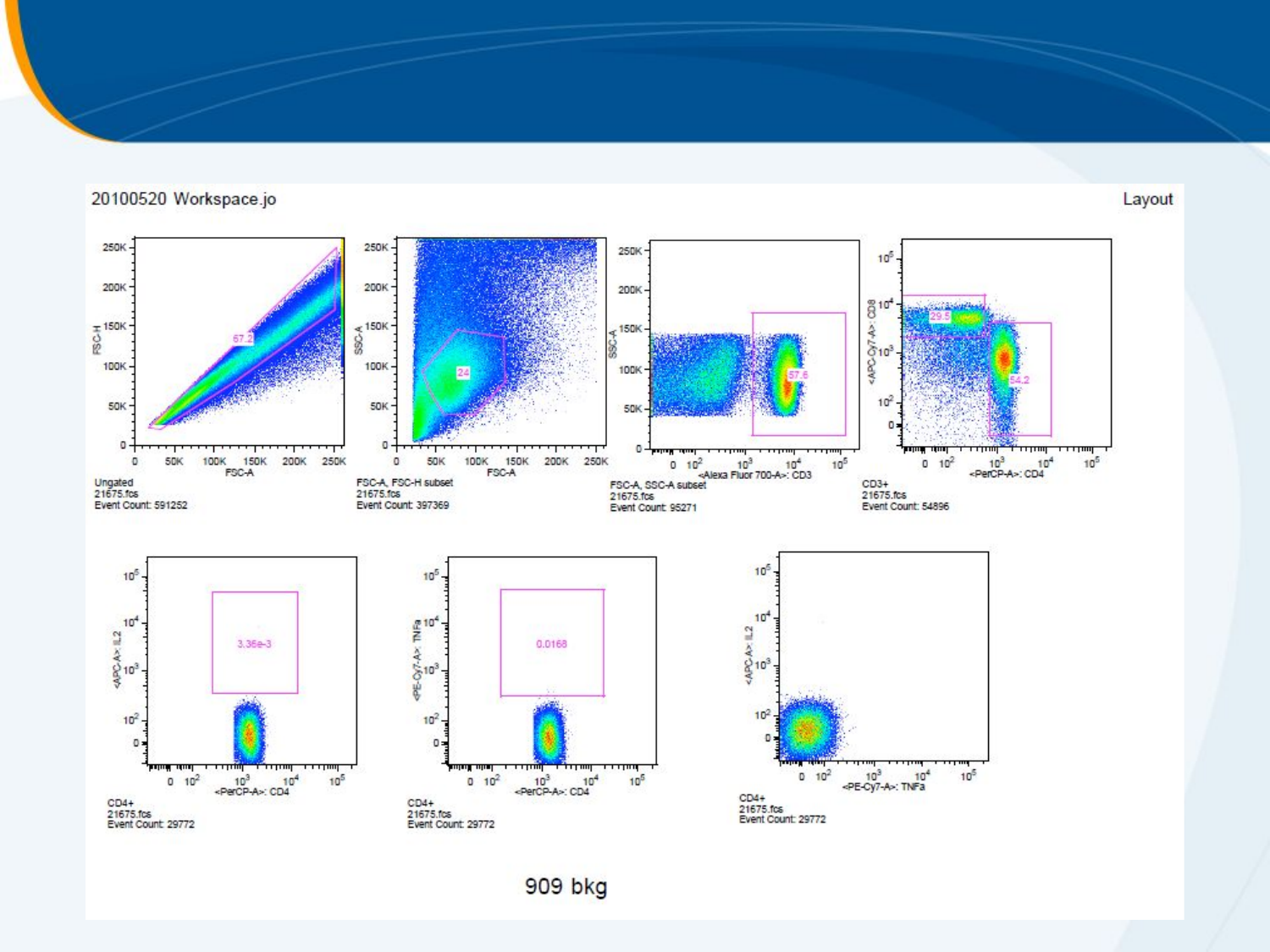

## Slide 2
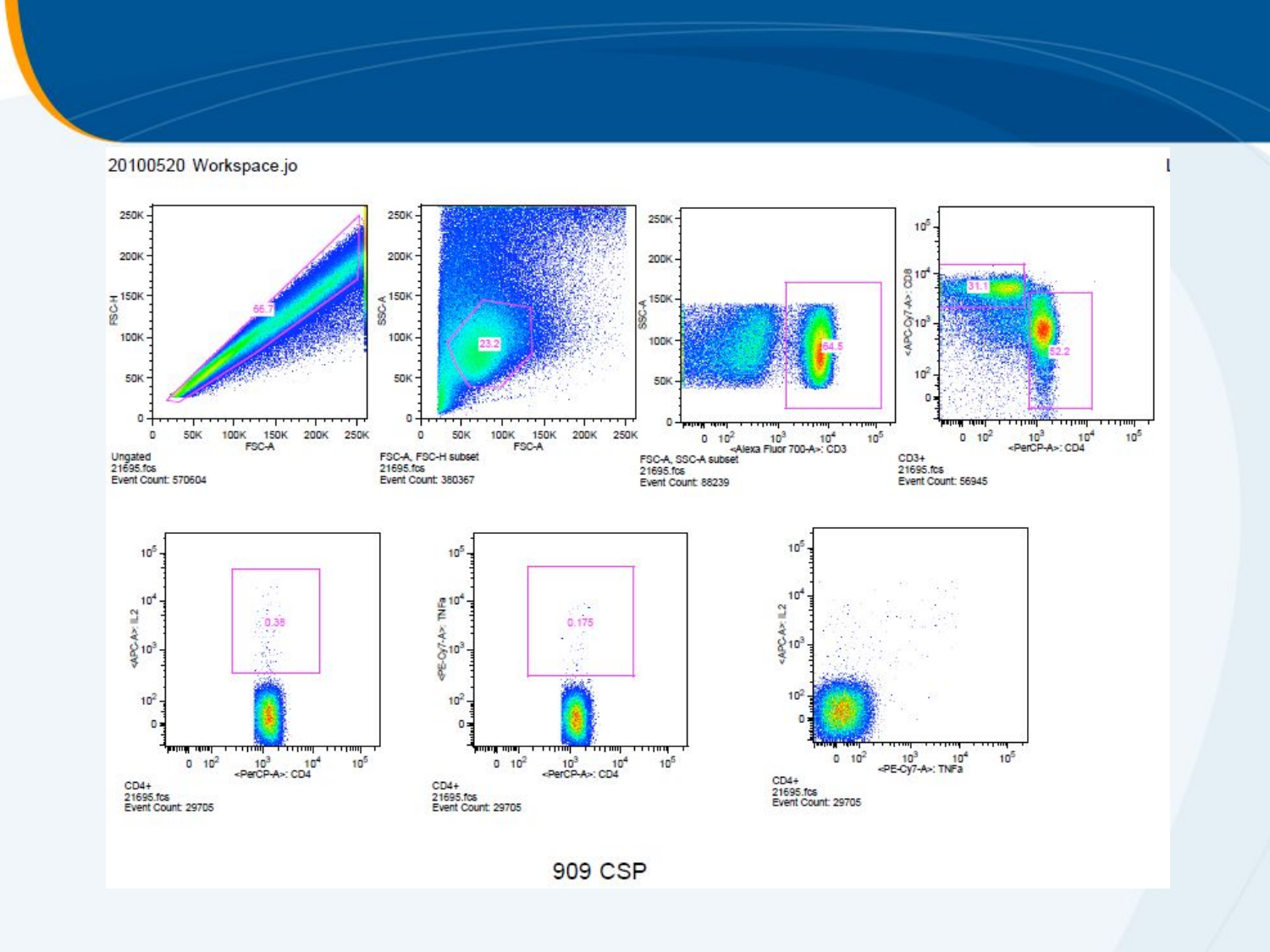

#

## Slide 3
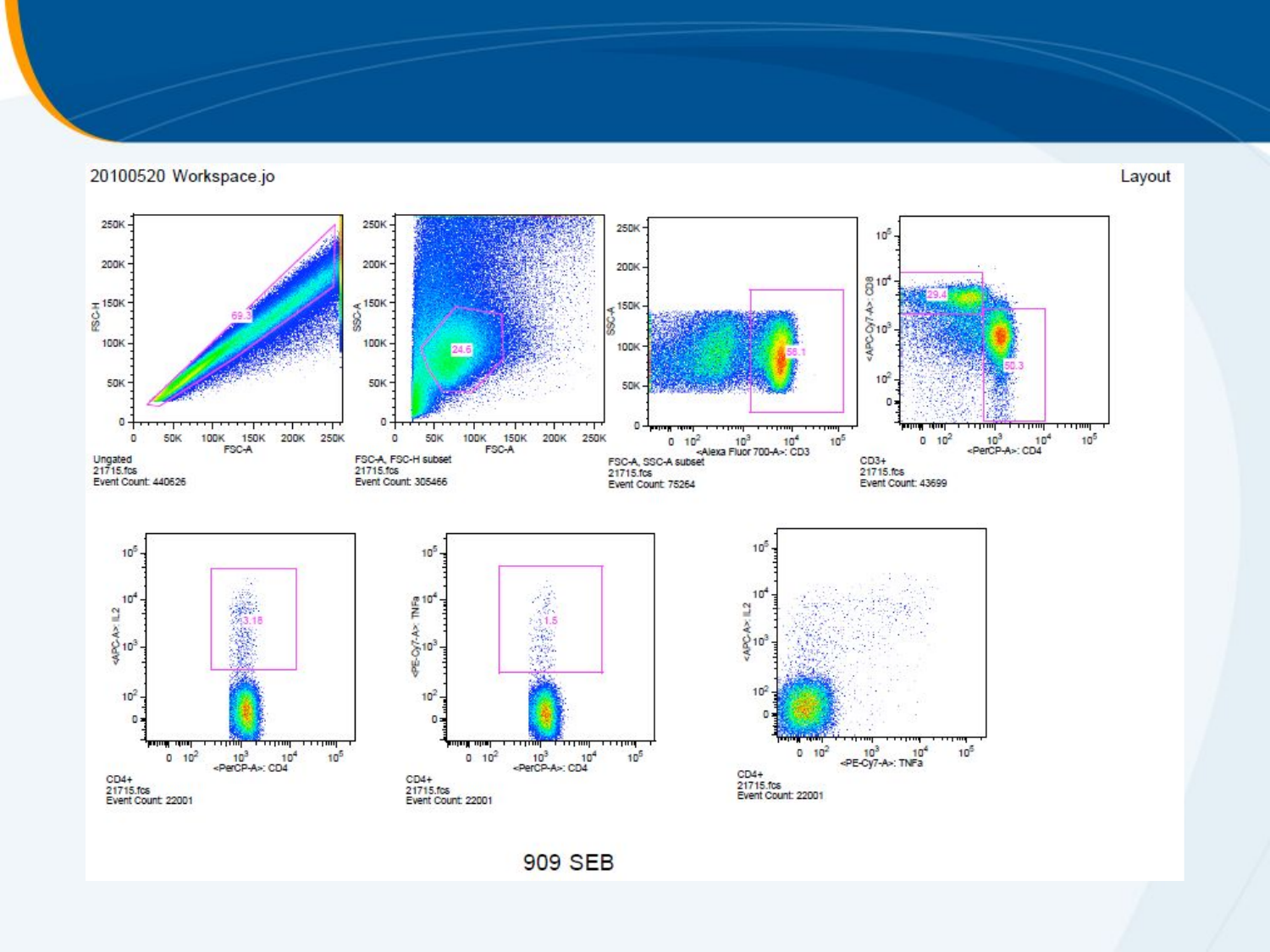

#

## Slide 4
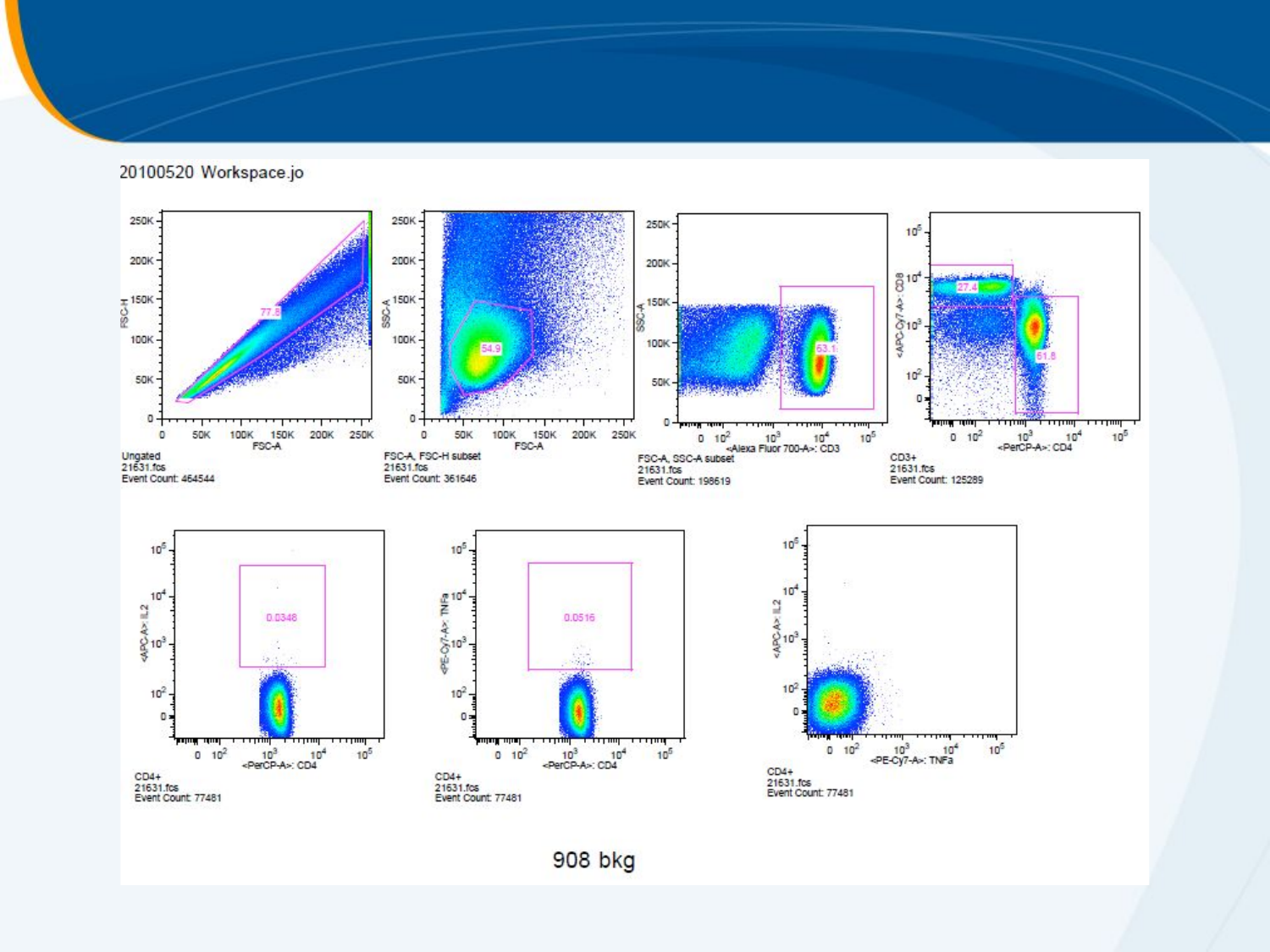

#

## Slide 5
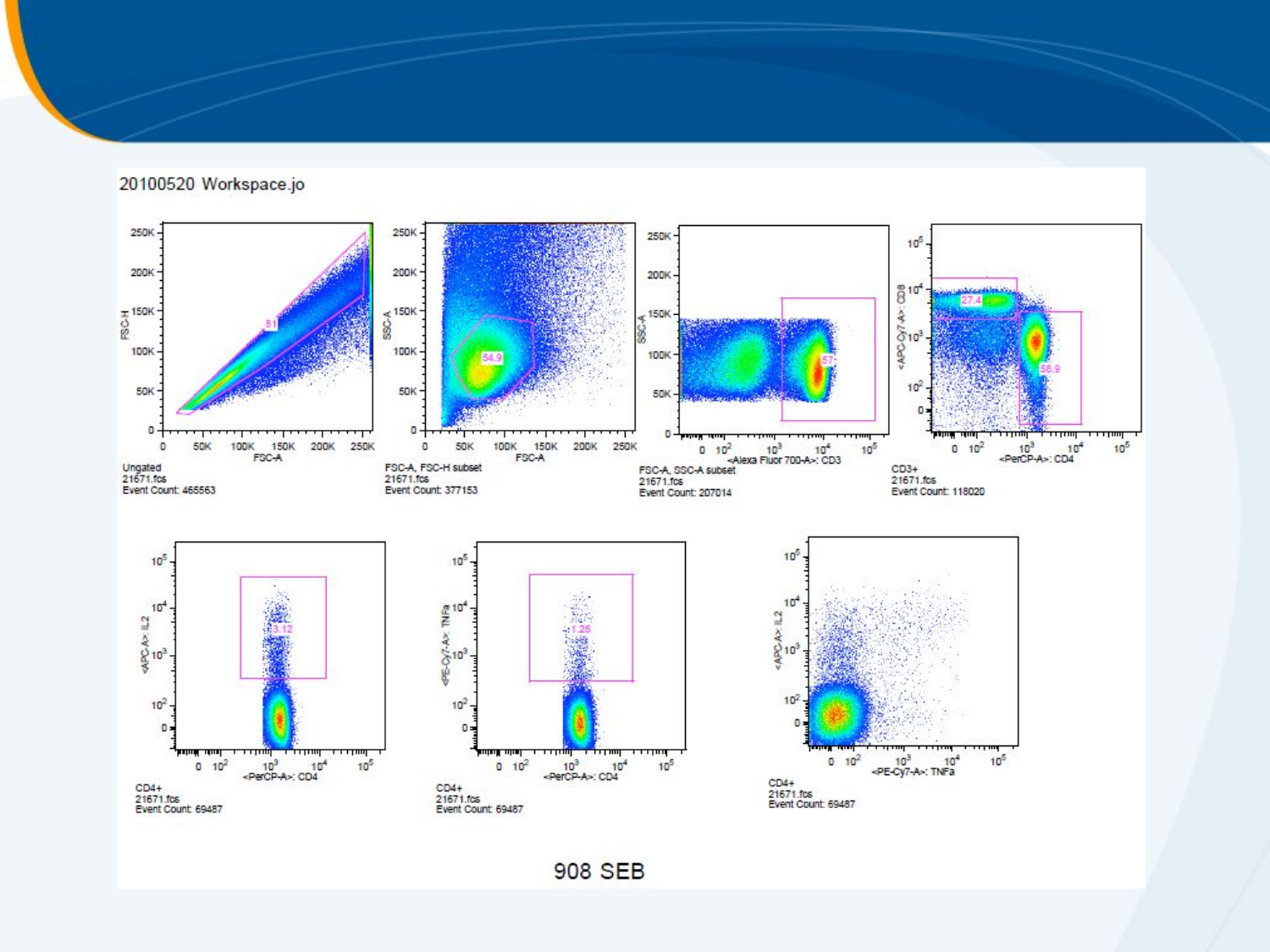

#

## Slide 6
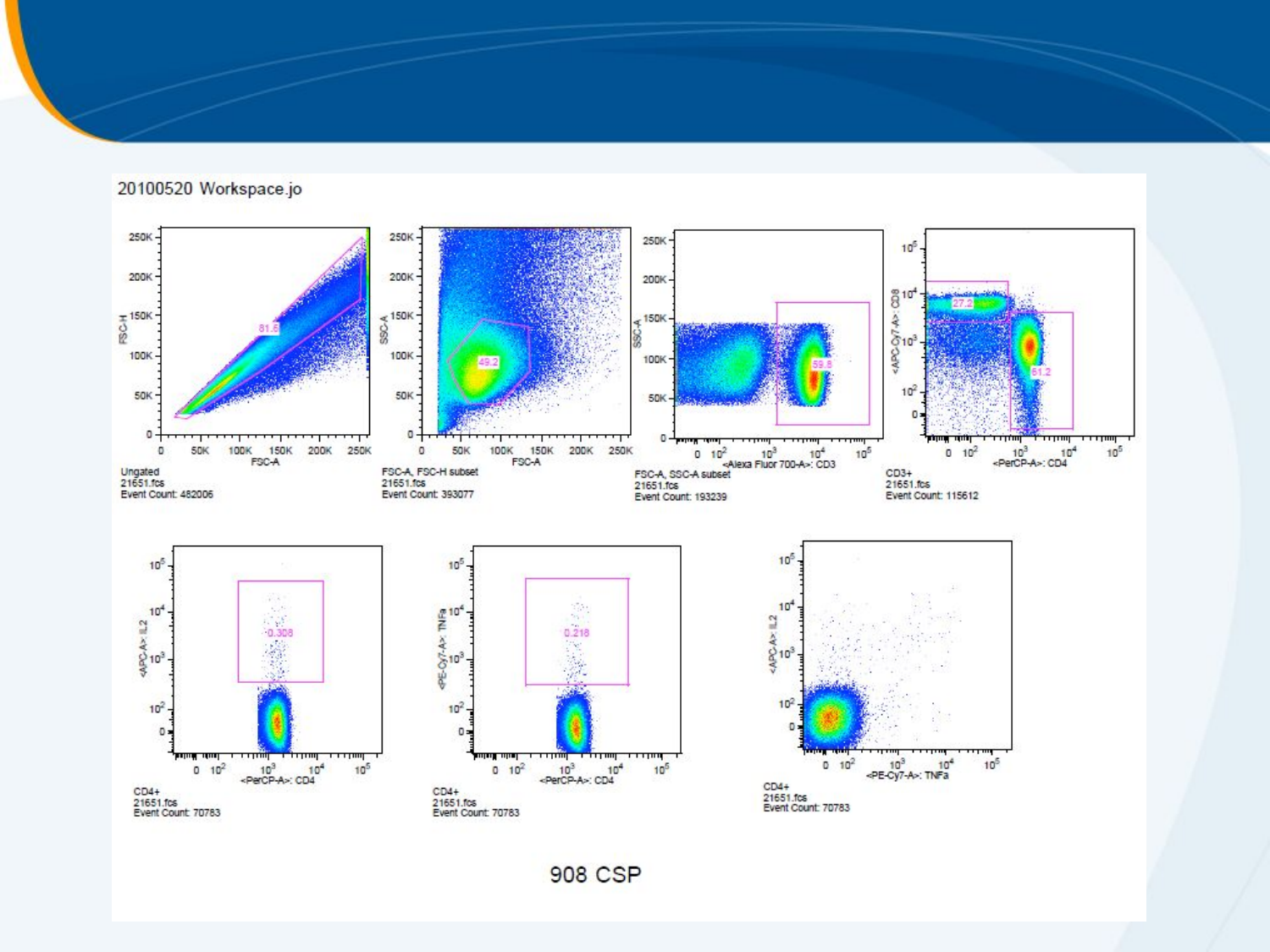

#

## Slide 7
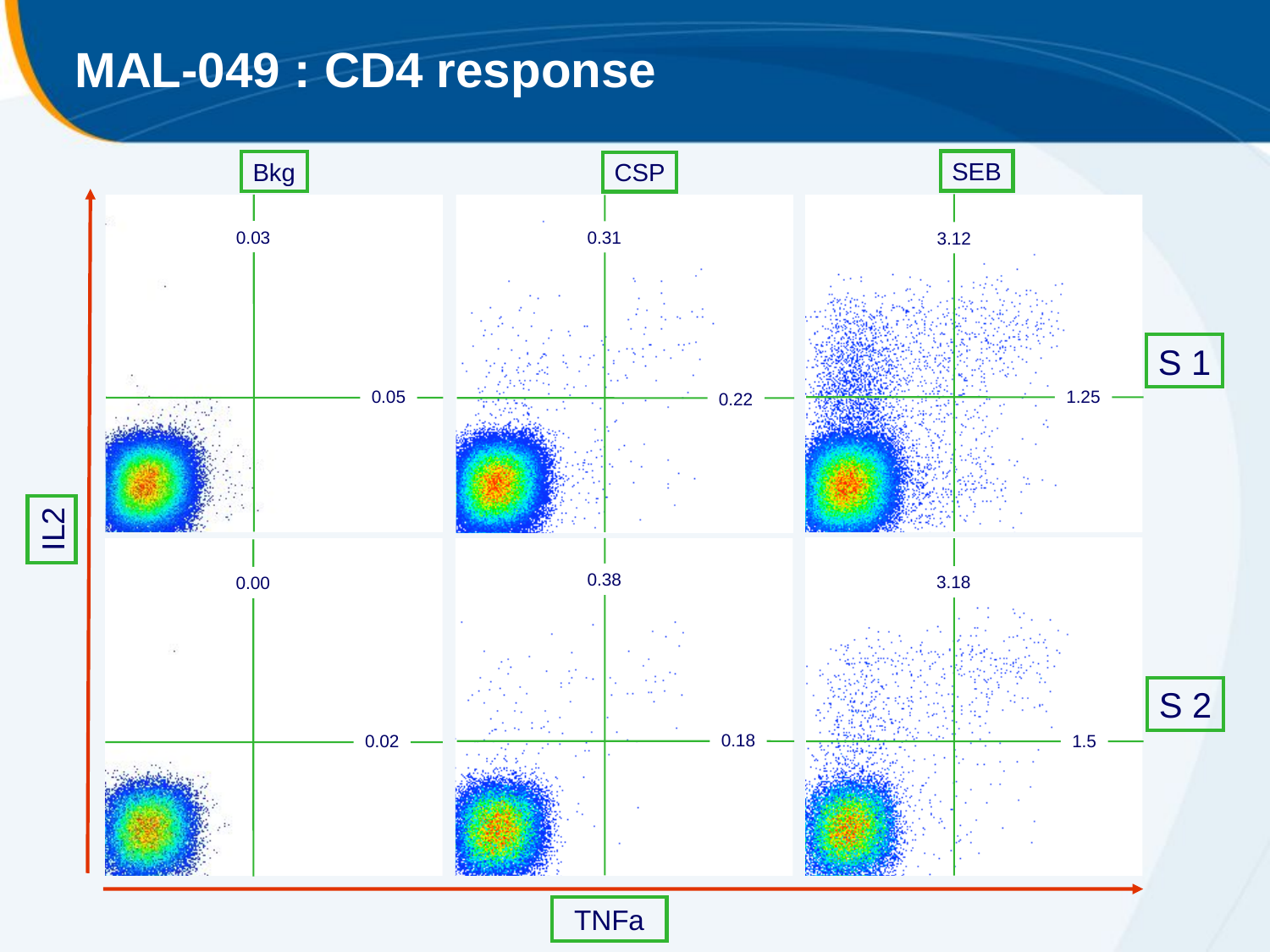

# MAL-049 : CD4 response
SEB
Bkg
CSP
S 1
IL2
S 2
TNFa
0.03
0.31
3.12
0.05
1.25
0.22
0.38
3.18
0.00
0.18
0.02
1.5

Supplement: Supporting Information S1 — (PPT) [file pone.0025786.s001.ppt]
